# Incidence of enamel defects on permanent canine teeth following extraction of linguoverted mandibular deciduous canine teeth in dogs

**DOI:** 10.3389/fvets.2024.1477179

**Published:** 2024-09-11

**Authors:** Charles L. Felz, Boaz Arzi, Kendall Taney, Katherine Block

**Affiliations:** ^1^Missoula Veterinary Dentistry and Oral Surgery, Missoula, MT, United States; ^2^Department of Surgical and Radiologic Sciences, University of California, Davis, Davis, CA, United States; ^3^Center for Veterinary Dentistry and Oral Surgery, Gaithersburg, MD, United States

**Keywords:** interceptive orthodontics, deciduous teeth, extraction, malocclusion, enamel defect, Turner’s tooth

## Abstract

Interceptive orthodontics may be indicated in puppies exhibiting dental malocclusion with linguoverted deciduous mandibular canine teeth to alleviate pain and prevent teeth interlock, which may affect growth and development of the mandibles. Historically extraction of deciduous mandibular canine teeth has been recommended as soon as a malocclusion is identified, often as early as 6–8 weeks of age and no later than 12 weeks of age. This early surgical intervention of deciduous teeth extractions risks potential damage to the developing permanent canine teeth resulting in enamel defects often referred to as a Turner’s tooth or Turner’s hypoplasia. A search of medical records from five veterinary specialty dentistry practices was conducted to identify dogs 8–12 weeks of age who (a) underwent deciduous mandibular canine extractions for management of class 1 or class 2 malocclusion with linguoverted mandibular canine teeth, and (b) were seen for at least one recheck exam to assess for enamel defects on permanent mandibular canine teeth. Furthermore, data was collected to determine the number of dogs that required additional treatment after eruption of the permanent canine teeth due to linguoversion of the permanent canine teeth. All procedures were performed by a board-certified veterinary dentist™ or a supervised veterinary dentistry resident. Seventy-four dogs fit the inclusion criteria and had a total of 143 deciduous mandibular canine teeth extracted, out of which 13 dogs exhibited enamel defects affecting 21 permanent canine teeth. The 13 affected dogs represent a 17.5% cumulative incident rate 13/74 (95%CI 11–28%). Of all extracted teeth, 14.6% (21/143) had enamel defects affecting permanent canine teeth. Twenty-eight dogs required additional treatment to prevent the permanent mandibular canine teeth from causing trauma to the hard palate and gingiva which represented 37.8% (28/74) of all dogs in the study. Age and sex of the dog at the time of extraction were not found to be associated with the likelihood of incidence of enamel defects. This is the first reported rate of enamel defects on permanent mandibular canine teeth following extraction of deciduous mandibular canine teeth and is important to consider when advising or performing extraction of deciduous teeth in dogs.

## Introduction

**Figure 1:**
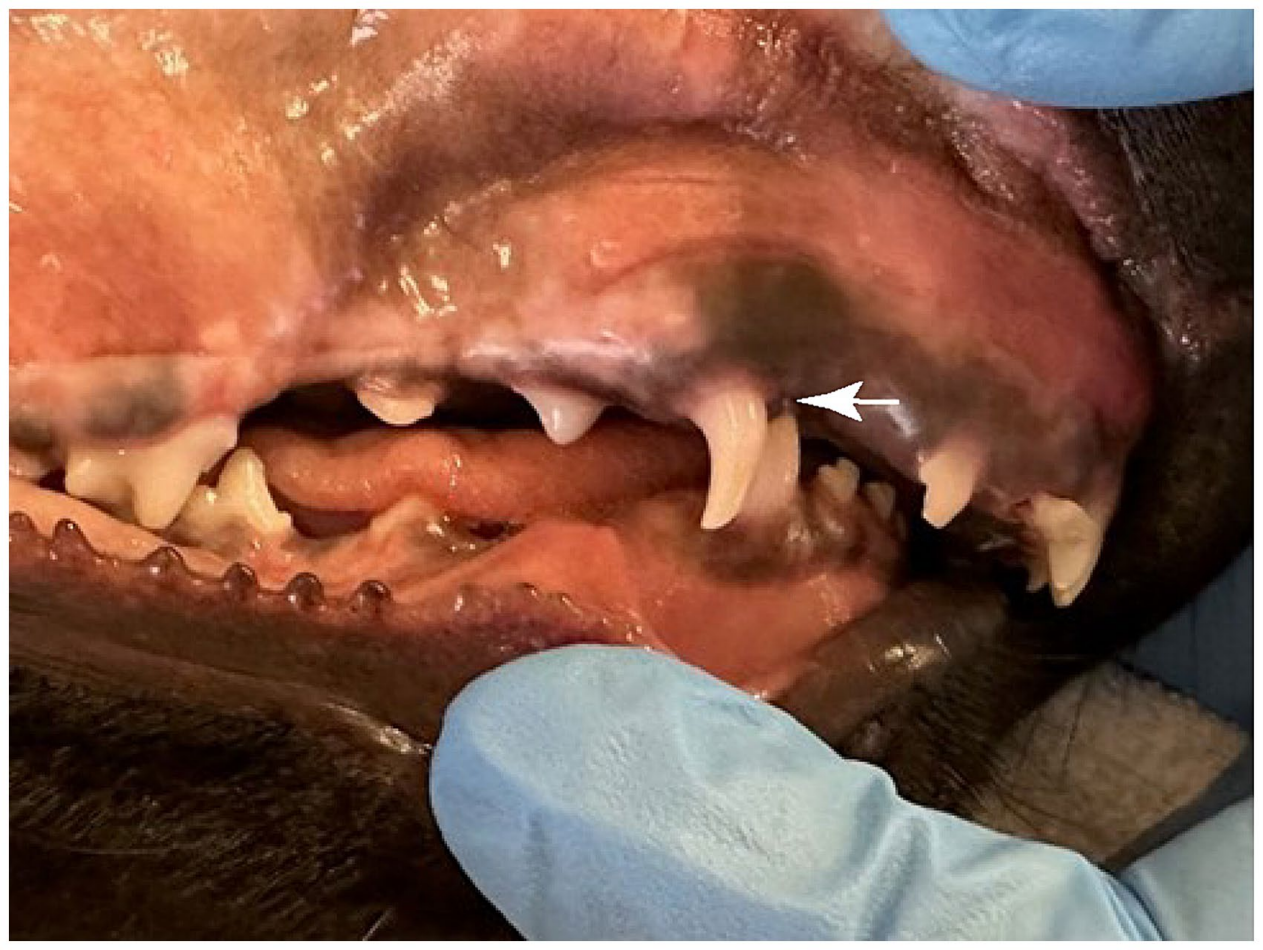
A class 2 malocclusion and linguoversion of the deciduous mandibular canine teeth (white arrow) in an 11-week-old dog resulting in trauma to the hard palate.

Interceptive orthodontics is a proactive orthodontic treatment that is presumed to allow for the development of normal permanent occlusion in puppies exhibiting malocclusion (1, 2). For this study classification of malocclusions were made according to American Veterinary Dental College (AVDC) Nomenclature. The AVDC defines a class 1 malocclusion as neutroclusion with normal rostrocaudal relationship of the maxillary and mandibular dental arches and malposition of one or more individual teeth and class 2 malocclusion as mandibular distoclusion with an abnormal rostrocaudal relationship between the dental arches in which the mandibular arch occludes caudal to its normal position relative to the maxillary arch (3). In this study interceptive orthodontics involved extraction of the deciduous mandibular canine teeth in patients with a class 1 or class 2 malocclusion and linguoverted canine teeth (Figure 1). Extraction of deciduous mandibular canine teeth should be performed with an abundance of caution to prevent damage to the developing permanent canine tooth. Given that enamel development ceases prior to or at the time of tooth eruption, enamel in dogs should be fully developed in permanent teeth 15–31.4 weeks after birth (4). This period of development makes enamel susceptible to injury from a variety of causes both environmental and systemic.

Most enamel defects are typically caused by trauma, infection, or inflammation resulting in a disruption during tooth development (4). Traumatic damage to the dental organ during development may result in enamel hypoplasia or hypocalcification, characterized by defects that are localized, irregular, and often affecting a single tooth (5). When enamel hypoplasia or enamel defects affect only one tooth they are known as a Turner’s tooth or exhibiting Turner’s hypoplasia (6). Likewise, systemic processes, such as fever, distemper, hypocalcemia, nutritional deficiencies, excessive intake of fluoride, and some drugs taken during the period of enamel formation, may result in enamel dysplasia. In these cases, all teeth developing at that time are affected.

**Figure 2:**
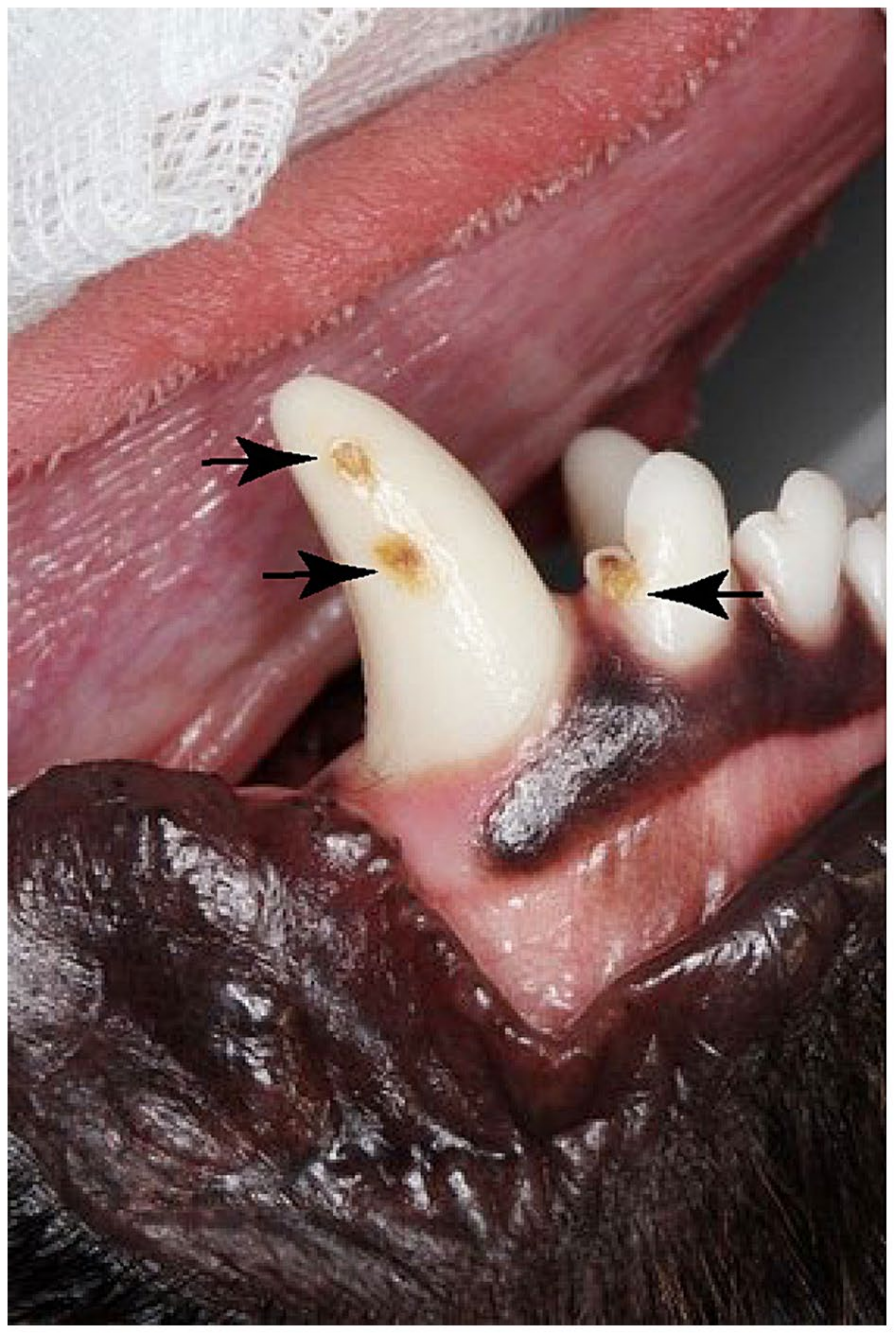
Enamel defects on a permanent right mandibular canine tooth and the right mandibular 3rd incisor (Black arrows).

**Figure 3:**
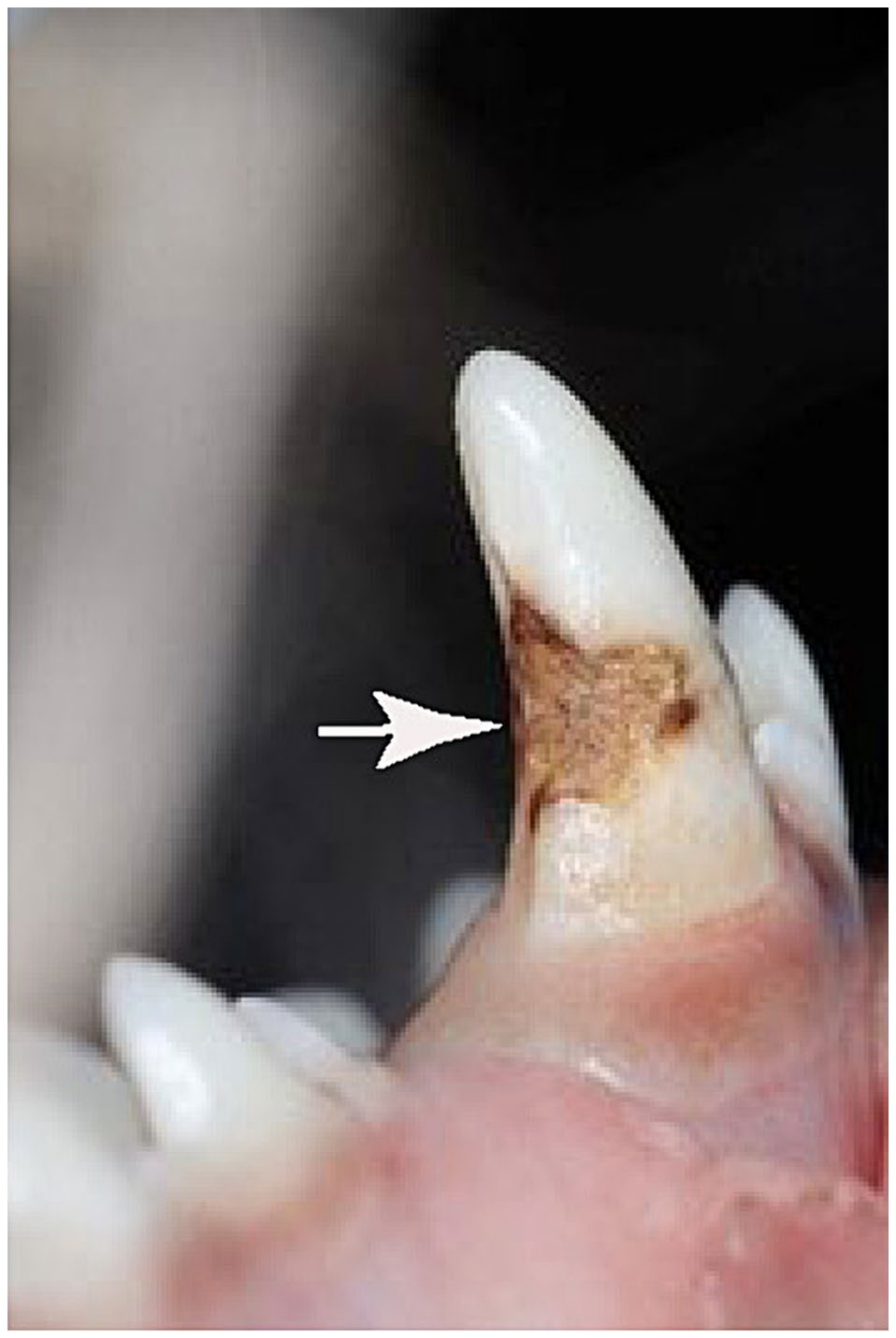
Enamel defect on a permanent right mandibular canine tooth (white arrow).

Hypoplasia is defined as a quantitative defect of enamel visually and is histomorphologically identified as an external defect involving the surface of the enamel and associated with reduced thickness of enamel (7, 8). The defective enamel may occur as shallow or deep pits or rows of pits arranged horizontally, or as small or large, wide or narrow grooves. Silberman et al. (9) created a simplified hypoplasia index (Table 1) which was used to characterize enamel hypoplasia of the affected teeth in this study. In the present study, all dogs had type-IV enamel hypoplasia due to trauma during extraction or post-operative inflammation resulting in brown and yellow enamel discoloration, abnormal coalescence, and missing enamel with the majority having defects on the lateral or occlusal aspect of the permanent canine teeth (Figures 2, 3).

**Table 1:** Simplified tooth hypoplasia index (9).

Type I hypoplasia: enamel discoloration due to hypoplasia
Type II hypoplasia: abnormal coalescence due to hypoplasia
Type III hypoplasia: missing some parts of enamel due to hypoplasia
Type IV hypoplasia: a combination of previous three types of hypoplasia

**Figure 4:**
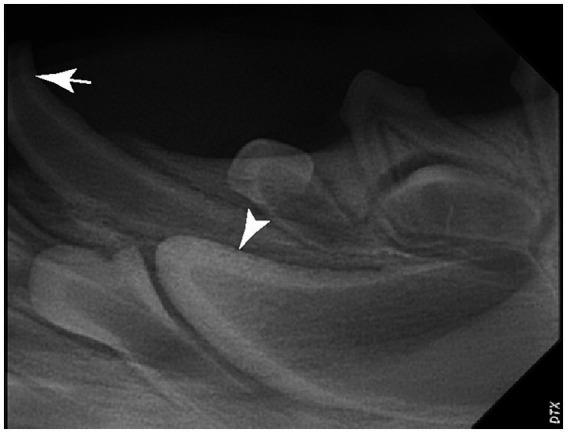
Dental radiograph of the left mandibular deciduous canine tooth (white arrow) and the developing permanent canine tooth (white arrowhead). Note the proximity of the apex of a deciduous mandibular canine to the crown of a developing permanent canine tooth.

The close anatomical relationship of the apex of the deciduous tooth to the developing permanent tooth bud explains the potential for possible developmental disturbances during extraction (Figure 4). A cephalometric study in people demonstrated the thickness of the hard tissue barrier between primary incisor teeth and their successors was <3 mm (10). However, no such data regarding the barrier between the deciduous mandibular canine teeth and the developing tooth buds exists for dogs which may have assisted in avoiding the potential disruptive effect of extractions on permanent tooth buds during odontogenesis. Deciduous canine teeth in dogs are long, thin, and fragile and operator technique during extraction may significantly traumatize the developing permanent tooth. Gentle tissue handling, and good hand control are essential while extracting the deciduous teeth. In addition, if a deciduous canine tooth fractures during extraction, removal of the root tip is crucial, because retained root tips may alter the eruption of the permanent tooth, and may lead to infection, further potentiating the formation of enamel defects. Prior to extracting a deciduous tooth, obtaining preoperative dental radiographs is essential to determine the shape and location of the root and to document the presence and location of the developing permanent tooth (11). Because of the close three-dimensional spatial anatomic relationship between the apex of primary teeth and the developing permanent tooth the use of a cone beam CT (CBCT) scan is beneficial in assessing more precisely the exact spatial relationship of the crown and the apex in relation to the permanent successor (12). Regardless of the extraction technique employed, post-extraction dental radiographs should be obtained to document complete removal of the deciduous canine and to potentially identify damage to the developing tooth bud.

The incidence and rate of enamel defects on permanent dentition following extraction of deciduous teeth in dogs has not been reported. Hence, the primary objective of this retrospective study was to determine an incident rate of enamel defects occurring following extraction of linguoverted mandibular deciduous canine teeth in young dogs which were causing palatal trauma, dental interlock, and signs of oral pain.

## Methods and materials

Medical records from five veterinary specialty dentistry and oral surgery practices were reviewed for dogs who underwent deciduous mandibular canine teeth extraction due to linguoverted canine teeth between January 2017 and December 2021. Dogs from 38 breeds included in the study were between 8 and 12 weeks of age at the time of surgery and had class 1 or class 2 malocclusions with linguoverted mandibular canine teeth. All dogs underwent surgical extractions of deciduous mandibular canine teeth (i.e., an open extraction technique) and returned for at least one in-person recheck exam to evaluate the permanent mandibular canine teeth. Dogs were excluded from the study if they were older than 12 weeks of age at the time of extraction, had deciduous incisor teeth extracted at the same time as the deciduous canine teeth, did not undergo an in-person recheck exam, or were lost to follow up. Patient information collected during original presentation included patient history, signalment, physical exam findings, oral exam findings including descriptions and photographs of the malocclusion. Information collected during recheck exams included written descriptions and photographs of enamel defects in permanent mandibular canine teeth and whether patients required additional treatment to prevent the permanent canine teeth from causing palatal and/or gingival trauma.

Surgical teeth extraction was performed as previously described (13). Briefly, a sulcular incision was made followed by raising a triangle mucosal flap using a periosteal elevator. Partial buccal alveolectomy was performed using a round burr on a high-speed handpiece followed by luxating the tooth using appropriate size luxator. Delivery of the tooth was done using extraction forceps (13). All surgical incisions were closed using an absorbable monofilament suture material in a simple interrupted fashion. Pre and post extraction radiographs were obtained for all patients included in this study.

### Statistical analysis

Statistical analyses were performed using SAS 9.4 (Cary, NC). A significance threshold of *p* < 0.05 was used. The assumption of normality for age was evaluated via inspection of QQ- and PP-plots, histograms, and skewness. Age was determined to not be normally distributed variables were summarized descriptively with median and interquartile range (IQR). Frequencies were reported as both numbers and percentages with 95% CIs where appropriate. Univariable logistic regression analyses were used to test for association of patient sex and age with odds of having an enamel defect. Log-likelihood *p*-values and odds ratios with profile-likelihood odds ratio confidence limits were reported.

## Results

The search of medical records from five veterinary dental specialty practices for dogs between the age of 8–12 weeks that underwent surgical extraction of deciduous mandibular canine teeth yielded 128 cases. Fifty-four cases were not reexamined following extraction and were not included in the study. Therefore, the final group of patients included in the study was 74 individual dogs where extraction of mandibular deciduous canine teeth was performed due to linguoverted mandibular canine teeth.

The 74 dogs included in the study represented 38 breeds with standard poodles (9.5%, 7/74), poodle and poodle crosses (including ‘doodle’ breeds) (21.6%, 6/74), and Labradors (6.7%, 5/74) being the most represented. Standard poodles, poodles, and poodle crosses were overrepresented accounting for 31% of the dogs included in the study. At the time of surgery, all dogs were sexually intact. There were 35 females and 39 males with a median (IQR) age of 12 (10–12) weeks at the time of tooth extractions for dogs that eventually did not exhibit enamel defects on permanent canine teeth and 10 (10, 11) weeks for dogs that did not exhibit enamel defects on permanent canine teeth following extraction. The age per week odds ratio (95%CI) was 0.72 (95%CI 0.48–1.07) (*p* = 0.098). Hence, based on the 95% confidence interval the odds of an enamel defect could decrease by as much 52% per week older an animal is or increase by as much as 7% per week older an animal is. This broad range demonstrates that while the dog’s age at the time of surgery was not significantly associated with the development of enamel defects, a clinically important association cannot be ruled out.

We noted that 10.4% (5/48) of dogs aged 11–12 weeks at the time of extraction and 30.7% (8/26) of dogs aged 8–10 weeks at the time of extraction subsequently developed enamel defects on the permanent canine teeth. The enamel defect rate was 20% (7/35) in female dogs and 15% (6/39) in male dogs [*p* = 0.603, female vs. male OR (95%CI) = 1.4 (0.4–4.7)]. There was no significant association between sex and odds of an enamel defect. In total 74 dogs had 143 teeth extracted, with 69 dogs having bilateral extraction of mandibular deciduous canines and 5 having unilateral extraction.

Of the 74 dogs who underwent surgical extraction, 13/74 had enamel defects on adult mandibular canine teeth 17.5% (95%CI 11–28%). In total there were 21 enamel defects noted on the permanent mandibular canine teeth of these 13 dogs which is an enamel defect rate of 14.6% (21/143). In addition, 37.8% (28/74) of dogs required additional treatment to prevent the permanent mandibular canine teeth from traumatizing the hard palate. These treatments included crown reduction and vital pulp therapy (82%, 23/28) and wedge gingivectomy (17.8%, 5/28).

## Discussion

Dogs exhibiting linguoversion of the deciduous mandibular canine teeth typically requires prompt treatment to alleviate pain, soft tissue trauma to the hard palate or gingiva, and to prevent or release any dental interlock if present (14). This practice is known as interceptive orthodontics and is often performed in young dogs. This is the first study to document the occurrence and rate of enamel defects that occurs due to damage to the developing teeth buds likely during extractions of deciduous canine teeth. We noted several clinically relevant findings. First, 17.5% of dogs had enamel defects on permanent mandibular canine teeth. Specifically, an incidence rate of 14.6% of permanent canine teeth exhibited enamel defects. In addition, the dog’s age at the time of extraction was not significantly correlated with the development of enamel defects. Finally, 37.8% of dogs required additional treatment to prevent the permanent mandibular canine teeth from traumatizing the hard palate.

Enamel defects may be genetic or environmental and often the exact etiology is unknown. Environmental or acquired enamel defects may be divided into those caused by local factors such as trauma, local inflammation or infection, and those caused by systemic factors such as prolonged fever, systemic infection such as distemper virus (15), excessive fluoride administration, and certain drugs. Specifically for the present study, a local factor was suspected when a single tooth or group of neighboring teeth were affected resulting in a Turner’s tooth or Turner’s hypoplasia. Unlike local occurrence, general systemic factors during development of the teeth may result in several or all the teeth being affected (i.e., semigeneralized or generalized occurrence). There are numerous hereditary, acquired, systemic and local etiological factors which are associated with enamel defects (16). Because enamel does not remodel, the defects theoretically present a record of the insults suffered by the enamel organ during development of the enamel. However, determining the specific timing of insults to the developing enamel is often difficult due to the current lack of knowledge regarding the chronology of the different stages of amelogenesis as well as individual variation in rates of enamel formation (16). The damage sustained by the permanent canine teeth in this study likely occurred during amelogenesis or the process of enamel formation during odontogenesis. Ameloblasts are the cells that produce enamel (17). Their life cycle is divided into six stages that include morphogenetic, organizing, formative, maturative, protective, and desmolytic. Enamel matrix is secreted in the formative stage whereas mineralization of the enamel matrix occurs in the maturation stage (18). During enamel maturation, a dynamic process with cellular, biochemical, genetic, and epigenetic changes takes place in the developing tissue (18). The developing dental enamel is highly susceptible to different systemic and local factors during the formative and maturative stages of amelogenesis. Due to the close anatomical proximity of the apex of the deciduous canine teeth to the developing permanent tooth bud, extraction of the deciduous mandibular canine teeth may damage the developing permanent teeth (19).

Damage resulting in a Turner’s tooth or Turner’s hypoplasia may occur acutely because of trauma by direct impact of the root of the deciduous tooth on the permanent tooth germ or by mechanical trauma related to instrumentation and improper extraction technique or because of inflammation or infection during the post-operative period (20). Although all the extractions in this study were performed with an open extraction technique, a closed extraction technique may be utilized when appropriate. Specifically, deciduous teeth that have undergone a substantial amount of root resorption as indicated on a pre-operative radiograph and may already be mobile may be amendable for closed extraction technique (i.e., non-surgical) (21). To the authors knowledge, it has not been reported if extraction technique influences the rate of enamel defects following extraction.

Inflammation and infection may play a role in the development of enamel defects post-operatively, there is no study in dogs linking extraction of deciduous teeth and the development of enamel defects due to post-operative inflammation or infection. One study in people found that if caries occurs in a primary tooth, the successor tooth is more than twice as likely to have an enamel defect and in the case of early tooth loss for reasons other than trauma such as extraction or infection, the permanent successor tooth was five times more likely to have an enamel defect (22). In the present study the role of post-operative inflammation vs. trauma to the developing permanent tooth germ during extraction leading to an enamel defect is unknown. There was no apparent post-operative infection and none of the dogs were treated with antibiotics. Hence, infection may not have been a contributing factor to the development of enamel defects in this study, however, this cannot be asserted.

We noted the dog’s age at the time of extraction was not significantly correlated with the development of enamel defects. The current published recommendation is to extract linguoverted deciduous mandibular canine teeth as soon as they are observed, which may be as early as 6–8 weeks of age (23). This recommendation was made to eliminate trauma to the soft tissues of the hard palate and gingiva, release dental interlock which may allow the mandibles to reach their full genetic potential, and to alleviate pain. Although age at the time of extractions and the development of enamel defects was not significant, the fact that 10.4% of dogs age11-12 weeks at the time of extraction and 30.7% of dogs age 8–10 weeks at the time of extraction subsequently developed enamel defects may suggest to wait on extracting deciduous mandibular canine teeth until the dog is older than 11 weeks to minimize the potential for damaging the developing permanent tooth bud. Importantly, it is unknown if a delay in performing extraction of mandibular deciduous canine teeth at 8–10 weeks of age until 11–12 weeks of age or older will affect the ability of the jaws to reach full genetic growth potential due to continued dental interlock and potential interference with skull development. A delay in extractions also has the potential for ongoing palatal trauma and patient discomfort. Timing of extractions is an area where further research is needed to provide a recommendation for the optimal time to perform extractions of deciduous canine teeth and minimize the occurrence of enamel defects in the permanent canine teeth as well as prevent dental interlock which may affect the ability of the jaws to reach their full genetic potential in both length and width (24).

Ultimately, when indicated, the extraction of deciduous teeth should be performed with great care and requires both proper extraction technique and carful operator technique. Pre- and post-extraction dental radiographs are essential when planning extractions to both identify the shape and location of the root and document the presence and location of the developing permanent tooth, as well as any damage present to the developing permanent tooth. The potential for damage to the tooth bud of developing permanent teeth should be discussed with the client prior to interceptive orthodontics (25). This should include the risks of negative sequelae associated with enamel defects such as increased plaque retention, weakened tooth structure, pulpitis, dentinal sensitivity, and endodontic disease. Finally, it should be noted that 37.8% of dogs required additional treatment to prevent the permanent mandibular canine teeth from traumatizing the hard palate. The possibility of additional treatment once the permanent canine teeth erupt should also be clearly communicated to the client.

Due to the retrospective nature of this study, evaluations of the 54 patients lost to follow up was not possible. It is possible that patients who were lost to follow up were perceived to be successful and/or functional in nature by their owners and primary care veterinarians and did not warrant further orthodontic or restorative intervention. This may have potentially altered the rate of enamel defects noted following extraction of the deciduous mandibular canine teeth.

## Conclusion

Extraction of linguoverted deciduous mandibular canine teeth is performed as an “interceptive” orthodontic procedure. However, we noted that those teeth extractions may result in enamel defects on the permanent teeth in 17.5% of dogs and an enamel defect rate of 14.6%. Informing clients that the potential for damage to the developing tooth bud may result in enamel defects on the permanent mandibular canine teeth is warranted.

## Data Availability

The original contributions presented in the study are included in the article/supplementary material, further inquiries can be directed to the corresponding author.
